# Reference gene selection for gene expression analysis in *Coffea arabica* L. under ABA and gibberellin treatments

**DOI:** 10.1007/s11033-026-12489-0

**Published:** 2026-07-31

**Authors:** Lillian Magalhães Azevedo, Robert Márquez-Gutiérrez, Matheus Martins Daúde, Horllys Gomes Barreto, Renato Ribeiro de Lima, Raphael Ricon de Oliveira, Antonio Chalfun-Junior

**Affiliations:** 1 Central Laboratory of Molecular Biology (LCBM), Institute of Natural Science (ICN), Federal University of Lavras (UFLA), Lavras, Minas Gerais Brazil; 2 Laboratory of Plant Molecular Physiology (LFMP), Plant Physiology Sector, Institute of Natural Science (ICN), Federal University of Lavras (UFLA), Lavras, Minas Gerais Brazil; 3 Laboratory of Molecular Analysis (LAM), Life Sciences Department, Federal University of Tocantins, Palmas, Tocantins Brazil; 4 Statistics Department, Federal University of Lavras (UFLA), Lavras, Minas Gerais Brazil

**Keywords:** Coffee plants, Gene expression, Reference genes stability, GA_3_ and ABA treatments

## Abstract

**Background:**

Quantitative reverse transcription polymerase chain reaction (RT-qPCR) requires stable reference genes (RGs) to ensure accurate target gene normalization. Plant growth regulators like abscisic acid (ABA) and gibberellins (GA_3_) play critical roles in plant reproductive transitions, yet validated RGs for *Coffea arabica* under these specific treatments remain scarce. This study aimed to evaluate the expression stability of eight candidate RGs in coffee leaves subjected to various hormonal concentrations.

**Methods and results:**

The stability of *CaACT*, *CaGAPDH*, *CaEFA1*, *CaTUB*, *CaAP47*, *CaMDH*, *CaRPL39*, and *CaUBQ2* was evaluated in leaf tissues of *Coffea arabica* during the reproductive stage under different ABA and GA_3_ treatments using five statistical algorithms: geNorm, NormFinder, BestKeeper, Delta-Ct, and RefFinder. The expression profiles of the candidate genes varied among treatments, with *CaEFA1*, *CaGAPDH*, *CaMDH*, and *CaACT* showing higher and more stable expression levels, whereas *CaTUB* and *CaAP47* were consistently ranked as the least stable genes. Pearson correlation analysis revealed high correlations among *CaRPL39*, *CaUBQ2*, *CaMDH*, *CaGAPDH*, *CaEFA1*, and *CaACT*, while *CaAP47* and *CaTUB* showed the lowest correlation coefficients. Validation analysis using the flowering-related gene *CaTFL1* demonstrated that normalization with stable RGs (*CaACT* and *CaMDH*) produced more consistent expression profiles and narrower confidence intervals than normalization using unstable RGs.

**Conclusions:**

*CaACT* and *CaMDH* are the most suitable RGs for RT-qPCR normalization in leaf tissues of *Coffea arabica* during the reproductive stage under ABA and GA_3_ treatments. These findings provide a reliable basis for future studies involving hormonal regulation and reproductive development in coffee plants and reinforce the importance of validating RGs for specific experimental conditions.

**Supplementary Information:**

The online version contains supplementary material available at 10.1007/s11033-026-12489-0.

## Introduction

Flowering in plants is tightly controlled by a complex interplay of hormonal and genetic signaling pathways that respond to both internal and environmental cues [1]. *Coffea arabica* L., a crop of significant economic importance, relies heavily on a thorough understanding of the mechanisms governing its reproductive development, particularly the flowering process, which initiates fruit formation [2]. In this crop, the asynchronous development of floral buds before and after reproductive induction results in prolonged and uneven harvest periods [3]. Plant growth regulators, such as abscisic acid (ABA) and gibberellins (GA), can significantly alter gene expression associated with the flowering process, modulating endogenous signaling pathways and physiological processes related to floral development [4]. Because the reproductive stage is one of the most critical phases influencing yield formation and production quality in crop species, studies investigating the molecular responses induced by ABA and GA treatments are highly relevant for understanding plant reproductive development.

ABA is classically known as the primary hormone related to abiotic stress responses, but it also plays a crucial role in regulating developmental processes, including bud dormancy and the floral transition [5]. ABA often acts antagonistically to growth-promoting hormones. Among these, gibberellins (GAs) are fundamental, actively promoting cell elongation, seed germination, and the induction of flowering in many species [6]. The interaction and balance between the levels of ABA (an inhibitor) and GAs (promoters) form a complex signaling pathway that integrates environmental and endogenous cues to determine the optimal time for flowering [4]. Therefore, the exogenous application of these two regulators represents a powerful model to dissect the gene networks governing reproductive development in the coffee plant.

In this context, the use of real-time PCR with reverse transcription (RT-qPCR), a highly sensitive technique used to quantify gene expression, is essential to investigate how hormones affect the gene regulation of flowering in coffee plants, enabling the development of more effective management strategies. The method involves the real-time amplification of cDNA, synthesized from an RNA template, which is monitored using fluorescent intercalating agents [7, 8]. The primary metric obtained is the quantification cycle (Cq), the cycle at which fluorescence exceeds a detection threshold, allowing for the determination of transcript quantity in the original sample [8, 9].

Accurate quantification of a target gene requires the use of normalization genes, which account for the variability introduced by the limitations of the available technology and preparation methods. This guarantees that fluctuations in the quantity of genetic material align with the variations detected in both the target gene and the control [10]. Additionally, the most commonly used methods for quantifying gene expression rely on the Cq values of reference gene (RGs) to determine the relative expression of target genes [11, 12]. Overall, a gene must meet two criteria to be considered a RG: it must be stably expressed under all conditions and have a level of expression above the background [13]. Typically, these normalization genes are housekeeping genes involved in essential cellular processes. Common examples, several of which are evaluated in this study, include those encoding *actin* (*ACT*), *glyceraldehyde-3-phosphate dehydrogenase* (*GAPDH*), *tubulin* (*TUB*) and *elongation factor 1α* (*EFA1*) [10].

To assess the reliability of such candidate genes, several well-established statistical algorithms have been developed, including geNorm, NormFinder, BestKeeper, and DeltaCt. These algorithms were used in this study along with the integrative tool RefFinder [13–18].

The stability of RGs in *Coffea arabica* has been previously investigated under a range of experimental contexts, including biotic stresses like fungal infection [19], various abiotic stresses such as drought [20, 21], salt, and heat [22], and elevated CO_2_ [23], as well as specific developmental processes like somatic embryogenesis [24]. Historically, candidate RGs selection in plants progressed from classical molecular methods such as Northern blotting and expressed sequence tags (ESTs) to modern high-throughput transcriptomics, including microarrays and RNA sequencing (RNA-Seq), often followed by robust statistical screenings [25]. Across these diverse conditions in coffee, genes such as *CaGAPDH* [19, 20, 22], *CaACT* [23, 24], and *CaMDH* [22, 23] have frequently been identified as stable normalizers. However, these studies collectively demonstrate that the ranking of the most stable genes varies significantly with the specific plant tissue and experimental conditions, highlighting the critical need for context-specific validation [19–24].

Although RGs have been validated for different tissues and conditions in coffee, this is the first study to focus on the key hormones (ABA and GA) involved in coffee flowering [4, 26]. Since ABA is associated with stress regulation and the reproductive transition [4, 27, 28] and GA_3_ influences growth and floral induction [29–31], we hypothesized that the expression of commonly used housekeeping genes would be unstable under these specific treatments. Accurate gene expression analysis during these processes requires reliable RGs, yet a consensus on the most suitable ones for this context is lacking. Therefore, the objectives of this study were: (1) to evaluate the stability of eight previously documented candidate RGs in coffee plants under different ABA and GA_3_ treatments using multiple statistical algorithms, and (2) to validate the most and least stable genes by normalizing the expression of the target gene, *CaTFL1*, to demonstrate the impact of proper RGs selection.

## Materials and methods

### Plant material and field conditions

The experiment was conducted at the Federal University of Lavras (UFLA), located in the municipality of Lavras, Minas Gerais, Brazil. The field is situated at an altitude of 918 m, with geographic coordinates of 21° 14’ 43” S latitude and 44° 59’ 59” W longitude. The plant material consisted of six-year-old *C. arabica* cv. Paraíso plants. The experimental design followed a randomized block design (RBD), with six treatments and two controls: water (control1), 0.05% (w/v) Tween 20 (control2), 5, 25, and 100 ppm GA_3_ in 0.05% (w/v) Tween 20, and 5, 25, and 100 ppm ABA in 0.05% (w/v) Tween 20. A preliminary analysis was conducted to assess the effect of the surfactant on gene expression. Since no significant differences were observed in the Cq values between the water-only control and the 0.05% (w/v) Tween 20 control, data from both groups were pooled for all subsequent analyses and are hereafter referred to as the “Control” group.

Each treatment consisted of three blocks (biological replicates) with each block containing three plants (experimental unit). The treatments were applied during the dry season and low-temperature period in this region, corresponding to August. To ensure uniformity of application, a fixed volume of 200 ml per plant was applied using a 12 L knapsack sprayer at 8:00 AM. The application was performed on the entire plant to ensure even distribution. For the GA_3_ treatments, Progibb 400^®^ (40% concentration) was used, while Ingrain^®^ (10% concentration) was used for the ABA treatments. Two hours after application, the leaves collected and immediately frozen in liquid nitrogen and stored at 80 °C until RNA extraction. The 2 h sampling time was selected based on previous studies demonstrating that early transcriptional responses to ABA and GA related signaling occur within this interval. Notably, Azevedo [4] reported significant changes in the expression of flowering-related genes in *Coffea arabica* leaves 2 h after the application of the same ABA and GA_3_ concentrations used in the present study. Similarly, previous research has shown that hormonal signaling pathways can trigger measurable gene expression changes within 2 h of treatment [32]. Therefore, this timepoint was chosen to capture primary transcriptional responses induced by the hormones while minimizing downstream secondary effects.

### Selection of target and reference genes

Eight potential RGs were selected based on previous RT-qPCR studies conducted in *C. arabica* [21, 23, 24]. These genes include those encoding *β-actin* (*CaACT*), *glyceraldehyde-3-phosphate dehydrogenase* (*CaGAPDH*), *elongation factor 1α* (*CaEF1Α*), *β-tubulin* (*CaTUB*), *clathrin adaptor protein medium subunit* (*CaAP47*), *malate dehydrogenase* (*CaMDH*), *60s ribosomal protein l39* (*CaRPL39*), and *ubiquitin-conjugating enzyme E2* (*UBQ2*). The selection of candidate genes was based on two primary criteria. The first was previously documented evidence of stable expression across a wide range of tissues and experimental conditions, including various abiotic stresses and developmental stages [21, 23, 24]. The second criterion was functional diversity, to minimize the risk of selecting co-regulated genes. This approach of selecting previously documented and functionally diverse candidates provided a robust panel for validation under the specific hormonal treatments of the present study.

The primers for the target gene *CaTFL1* were designed specifically for this study. The transcript sequence for *CaTFL1* was obtained from the National Center for Biotechnology Information (NCBI) database. Primer design was performed using the Primer-BLAST tool available at NCBI. The parameters were set to yield an amplicon size between 100 and 200 bp, with primer melting temperatures (Tm) of approximately 60 °C and a GC content between 40 and 60%. The specificity of the designed primers was verified in silico via BLAST analysis against the *Coffea arabica* genome to ensure no potential off-target binding. The final primer sequences are listed in Table 1.

**Table 1:** Primer sequences and amplicon characteristics of the eight candidate reference genes and the target gene evaluated for RT-qPCR analyses in leaf tissues during the reproductive stage of *Coffea arabica* under ABA and GA_3_ treatments

Primer name	Sequence 5’-3’	Gene	Article	Efficiency (%)	*R*²	Amplicon size (bp)
RPL39_Fw	GCGAAGAAGCAGAGGCAGAA	60 S ribosomal protein L39	[21]	99	0.99	80
RPL39_Rv	TTGGCATTGTAGCGGATGGT					
UBQ2_Fw	GATGATACTTGGCCCTGCAC	ubiquitin-conjugating enzyme E2	[23]	99	0.99	145
UBQ2_Rv	CCTTCCCAGCTTGTCAATGT					
MDH_Fw	CCTGATGTCAACCACGCAACT	Malate Dehydrogenase	[23]	99	0.99	101
MDH_Rv	GTGGTTATGAACTCTCCATTCAACC					
AP47_Fw	GGTGTACGCTCACCATTTTCATC	clathrin adaptor protein medium subunit	[21]	98	0.98	82
AP47_Rv	AGCCAACAGCACCAGTAACTTG					
TUB_Fw	TCGGGCTGTCCTCATGGAT	*β*-tubulin	[21]	103	0.99	84
TUB_Rv	TTGTCGGGCCTGAAGATCTG					
ACT_Fw	AAGCTTGCCTATGTGGCTCTTG	Actin	[23]	101	0.99	100
ACT_Rv	TCACTTGTCCATCTGGCAATTC					
GAPDH_Fw	GGGAAGAGCTGCTTCATTTAACA	Glyceraldehyde 3-phosphate dehydrogenase	[21]	98	0.98	84
GAPDH_Rv	CCATTGAGGGCTGGAAGAAC					
EFA1_Fw	GGTGGTTTTGAAGCTGGTATTTCT	Elongation factor 1α	[21]	102	0.99	100
EFA1_Rv	TGTTGCAGCAGCAGATCATTT					
TFL1_Fw	GTTTTCTGCTAGCTTCGACA	TERMINAL FLOWER1	This study	101	0.98	89
TFL1_Rv	TTGGAGTGAAGCTGTCTACC					

### RNA extraction and cDNA synthesis

For each biological replicate, a total of 12 fully expanded leaves were collected, comprising four leaves from the middle third of each of the three plants per experimental plot. These leaves were pooled to represent a single experimental unit prior to RNA extraction. Total RNA was then extracted from 200 mg of this pooled and ground tissue using the protocol described by de Oliveira [33]. After determining the RNA concentration and quality, 5 µg of RNA was treated with DNase I using the Turbo DNA-free Kit (Ambion). The RNA quality and quantity were evaluated using a NanoVue^®^ spectrophotometer (NanoVue GE Healthcare). The integrity of the RNA was verified using agarose gel electrophoresis and visualized on a UV-transilluminator photodocumentator (UVITEC FireReader XS D-77Ls-20.M). cDNA was synthesized from 1 µg of treated RNA using the High-Capacity cDNA Reverse Transcription Kit (Applied Biosystems^®^), according to the manufacturer’s recommendations.

### RT-qPCR assay

The RT-qPCR assay was conducted using three biological and two technical replicates for each sample. Stock cDNA from the reverse transcription reaction was first diluted 1:3 (v/v) with nuclease-free water. Each reaction consisted of 1.5 µL of cDNA, 7.5 µL of SYBR (QuantiFast SYBR Green PCR Kit – Qiagen), 1.5 µL of each primer (10 µM), and 3.0 µL of RNase-free water, totaling 15 µL of final volume. The amplification conditions consisted of an initial activation step at 95 °C for 5 min, followed by 40 cycles of denaturation at 95 °C for 5 s and annealing/extension at 60 °C for 10 s, which is optimized for fast-cycling chemistries combined with short amplicon sizes (Table 1). To verify primer specificity, melting curves were generated after completing the 40 amplification cycles by gradually increasing the temperature from 60 °C to 95 °C. A single peak was observed for all primer pairs, confirming specific target amplification and the absence of nonspecific products or primer dimer formation (Supplementary Figure S1). Furthermore, standard curves generated from serial cDNA dilutions showed amplification efficiencies within the acceptable range and high linearity (R² values), validating the performance of all primer pairs (Supplementary Figure S2). For this experiment, RT-qPCR parameters were followed according to the Minimum Information for Publication of Quantitative Real-Time PCR Experiments (MIQE) guidelines, as detailed in Supplementary Table S1 [34].

### Expression stability assay

The quantification cycle (Cq) values were used to assess the stability of eight candidate RGs (*CaACT*, *CaGAPDH*, *CaUBQ2*, *CaMDH*, *CaEFA1*, *CaRPL39*, *CaTUB*, *CaAP47*), using five different statistical algorithms. geNorm calculates a variability coefficient by considering the expression variation of a candidate gene and its relation to other candidate genes [14]. Meanwhile, NormFinder takes into account both intra- and inter-group variations and is ideal for determining RGs to compare different conditions, such as in multifactorial experiments [13]. For its part, BestKeeper evaluates the stability of Cq values of a normalization gene across different conditions, and therefore, the standard deviation must be low for a gene to be considered a candidate by BestKeeper [15]. The DeltaCt method uses the differences in Cq values between conditions and samples, asserting that a candidate gene should have a maximum value of 0.5 when the difference is calculated [16]. These four tools have been integrated into a single tool, RefFinder, which calculates a coefficient value by considering the parameters from the other tools [17, 18].

To evaluate the stability of the candidate RGs, the following sample sets were used: leaves from controls and ABA-treated plants, leaves from controls and GA_3_-treated plants, and all the samples studied. Other combinations of the evaluated samples can be accessed through the Cq values available in the supplementary material (Supplementary Table S2) and, after the publication of the article, will be available for analysis in the RGeasy database [35].

### Validation of reference genes

Expression analysis of the *TERMINAL FLOWER* gene (*CaTFL1*) was performed to validate the RGs in coffee leaves subjected to treatments with three different concentrations of ABA or GA_3_, as well as untreated control plants. To demonstrate the impact of reference gene selection, the expression level of *CaTFL1* was normalized using two distinct pairs of genes: the pair identified as most stable (*CaACT* and *CaMDH*) and the pair identified as least stable (*CaAP47* and *CaTUB*). The expression rate and the confidence intervals were calculated considering the linear mixed-effect model [36] with lmer4 package [37]. Prior to model fitting, the assumptions of normality of residuals and homogeneity of variance were evaluated through residual diagnostic analyses. The graphics were generated in R (R Core Team, 2024) using the ggplot2 package [38].

## Results

### Expression levels of candidate reference genes

**Fig. 1:**
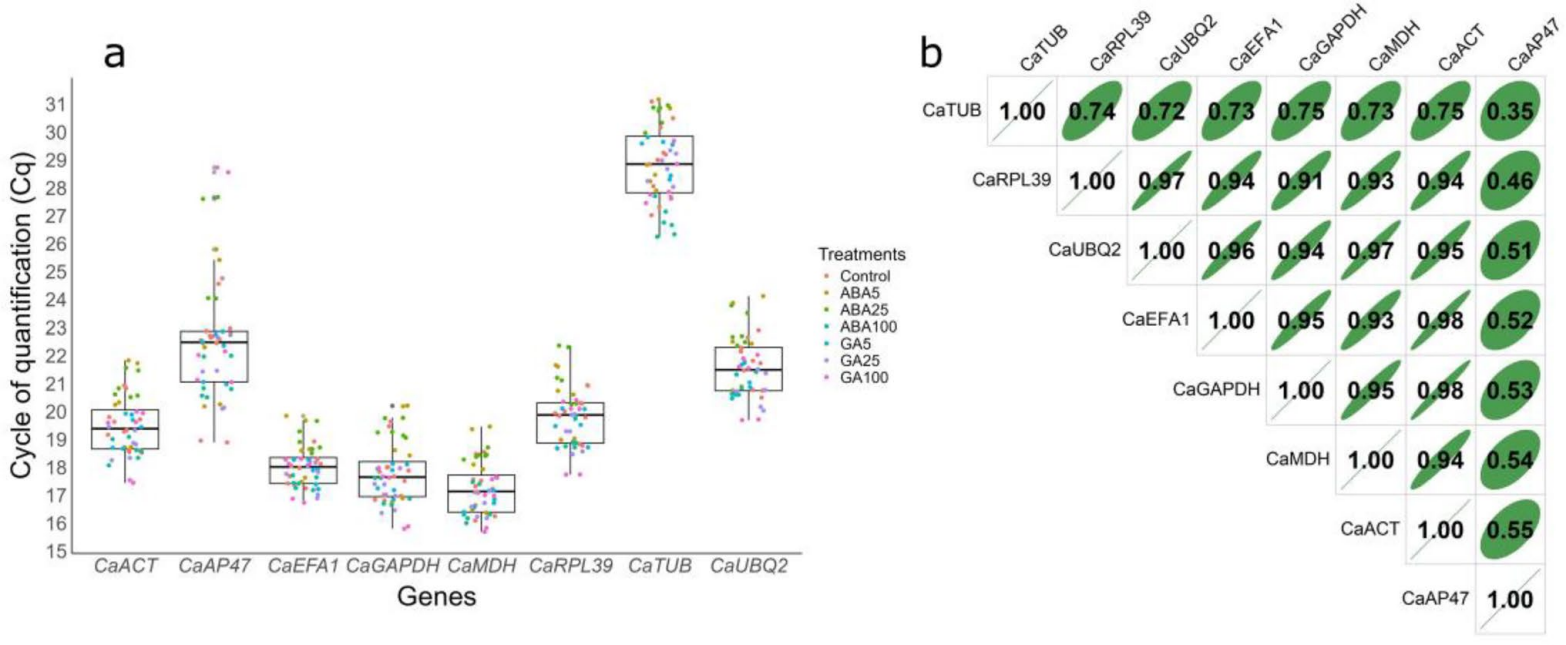
Distribution of quantification cycle (Cq) values and transcriptional correlation of the eight candidate reference genes evaluated in *Coffea arabica* leaves under hormonal treatments. (**a**) Boxplot representation of the raw Cq values across all samples treated with different concentrations (5, 25, and 100 ppm) of ABA and GA_3_, including untreated control plants. The black horizontal line inside each box indicates the mean value; the box margins represent the 25th (lower) and 75th (upper) quartiles; the whiskers indicate the 10th and 90th percentiles. Individual data points are colored according to specific hormonal conditions to illustrate data distribution and expression heterogeneity. (**b**) Pairwise Pearson correlation matrix of the candidate RGs based on their Cq profiles. Numeric values within the matrix represent the Pearson correlation coefficients (***r***); the shape, elongation, and orientation of the green ellipses visually reflect the strength of the correlation, where highly elongated ellipses indicate tighter, coordinated expression patterns (***r*** ≥ 0.91) and rounder shapes indicate uncoordinated transcript variations

Eight potential RGs were evaluated in coffee plants treated with three concentrations of GA_3_ and ABA. The raw quantification cycle (Cq) values for these genes, which are inversely proportional to transcript abundance (i.e., lower Cq indicates higher abundance), are presented in Fig. 1a. It can be observed that *CaTUB* exhibited the lowest expression level compared to the other seven genes, with an average Cq value around 29. The genes with mid-level expression included *CaAP47*, *CaUBQ2*, *CaACT*, and *CaRPL39*, with average Cq values ranging from 19 to 23. Finally, the genes with the highest expression levels were *CaEF1*, *CaGAPDH*, and *CaMDH*, with average Cq values ranging between 17 and 18. Moreover, these last three genes showed a similar expression pattern with greater data homogeneity, unlike *CaAP47* and *CaTUB*, which exhibited more variability in their data. Overall, treatment with 25 ppm of ABA decreased the expression of all eight genes. The gene with the highest homogeneity was *CaEFA1*, with Cq values ranging from 16.73 to 19.84, followed by *CaACT*, with Cq values ranging from 17.44 to 21.93, and *CaMDH*, which had Cq values between 15.69 and 19.69. Meanwhile, the gene with the highest heterogeneity was *CaAP47*, with Cq values ranging from 18.89 to 28.73, followed by *CaTUB*, with Cq values ranging from 26.45 to 31.17. These data demonstrate the variation in the expression of the candidate RGs, based on the Cq values, but stability was assessed using five algorithms.

To assess the correlation between the expression profiles of these eight genes across all samples, a Pearson correlation analysis was conducted to estimate the relationships among them. According to the pairwise correlation analysis, a group of six genes (*CaRPL39*, *CaUBQ2*, *CaMDH*, *CaGAPDH*, *CaEFA1*, and *CaACT*) exhibited high correlation with each other, with values ranging from 0.91 to 0.98. This indicates their expression patterns were highly similar across the different experimental conditions. In contrast, the genes *CaAP47* and *CaTUB* showed the lowest correlation coefficients, both when compared to the other genes and to each other, with values ranging from 0.35 to 0.75 (Fig. 1b). This suggests their expression patterns were distinct and not coordinated with the other candidate genes.

### Stability rankings of reference genes

With the aim of assessing the stability of each RGs in coffee plants treated with ABA and GA_3_, expression variability was analyzed using five established algorithms to rank each gene. In control plants and those treated with three concentrations of ABA, it was observed that, according to the geNorm tool, the most stable genes were *CaACT* and *CaGAPDH*, each with an M value of 0.177, followed by *CaUBQ2* and *CaMDH* (Table 2). According to the NormFinder tool, the ranking classified the most stable genes as follows: *CaACT*, *CaGAPDH*, and *CaUBQ2*, with SV values of 0.073, 0.170, and 0.243, respectively. With regard to the BestKeeper ranking, the most reliable genes were *CaEFA1*, *CaMDH*, and *CaRPL39*. In the case of the Delta-Ct ranking, the most stable genes were *CaACT*, *CaGAPDH*, and *CaUBQ2*, similarly to the RefFinder tool. In all cases, the least suitable genes to use as references in coffee plants treated with ABA were *CaTUB* and *CaAP47*, which were consistently ranked last (Table 2).

**Table 2:** Ranking of candidate reference genes according to stability values calculated by the geNorm, NormFinder, BestKeeper, Delta-Ct, and RefFinder algorithms, using Cq (Quantification Cycle) data obtained from leaves of plants treated with ABA. M, mean expression stability value (geNorm); SV, stability value (NormFinder); SD, standard deviation of Cq values (BestKeeper); ΔCt, ΔCt stability value (comparative ΔCt method)

Gene	geNorm M	Rank	NormFinder SV	Rank	BestKeeper SD	Rank	ΔCt	Rank	RefFinder	General Rank
*CaACT*	0.177	1	0.073	1	0.987	5	0.568	1	1.495	1
*CaGAPDH*	0.177	1	0.170	2	1.032	6	0.591	3	2.449	2
*CaUBQ2*	0.248	2	0.243	3	0.926	4	0.591	2	2.913	3
*CaMDH*	0.274	3	0.300	4	0.862	2	0.622	4	3.364	4
*CaEFA1*	0.337	5	0.433	6	0.671	1	0.685	6	3.834	5
*CaRPL39*	0.307	4	0.380	5	0.906	3	0.650	5	4.401	6
*CaTUB*	0.511	6	0.826	7	1.421	7	1.039	7	7.000	7
*CaAP47*	0.803	7	1.627	8	1.533	8	1.678	8	8.000	8

Using the same tools but analyzing expression data from coffee leaves treated with GA_3_, it was observed that geNorm ranked the genes from most to least stable as follows: *CaMDH* and *CaUBQ2* (both with the same M value), followed by *CaACT* (Table 3). Meanwhile, according to NormFinder, the most suitable genes were *CaACT*, *CaMDH*, and *CaRPL39*. According to the BestKeeper tool, however, the most stable genes were *CaEFA1*, *CaMDH*, and *CaGAPDH*. Both Delta-Ct and RefFinder showed similar rankings, with *CaMDH*, *CaACT*, and *CaUBQ2* being the most stable genes. Overall, across all tools used, the least stable genes were *CaTUB* and *CaAP47*, as similarly observed in the ABA treatment (Table 3).

**Table 3:** Ranking of candidate reference genes according to stability values calculated by the geNorm, NormFinder, BestKeeper, Delta-Ct, and RefFinder algorithms, using Cq (Quantification Cycle) data obtained from leaves of plants treated with GA_3_. M, mean expression stability value (geNorm); SV, stability value (NormFinder); SD, standard deviation of Cq values (BestKeeper); ΔCt, ΔCt stability value (comparative ΔCt method)

Gene	geNorm M	Rank	NormFinder SV	Rank	BestKeeper SD	Rank	ΔCt	Rank	RefFinder	General Rank
*CaMDH*	0.236	1	0.118	2	0.541	2	0.628	1	1.414	1
*CaACT*	0.318	3	0.087	1	0.648	4	0.640	2	2.378	2
*CaUBQ2*	0.236	1	0.174	4	0.691	5	0.646	3	2.783	3
*CaEFA1*	0.336	4	0.244	5	0.461	1	0.677	5	3.344	4
*CaRPL39*	0.265	2	0.169	3	0.709	6	0.660	4	3.834	5
*CaGAPDH*	0.354	5	0.295	6	0.634	3	0.688	6	5.045	6
*CaTUB*	0.485	6	0.882	7	0.859	7	1.043	7	7.000	7
*CaAP47*	0.882	7	2.041	8	1.337	8	2.071	8	8.000	8

With the aim of selecting the most suitable RGs for an experiment involving both ABA and GA_3_ treatments, the Cq values from all treatments were analyzed using the same tools as before. It can be seen that geNorm classified *CaACT*, *CaGAPDH*, *CaUBQ2*, and *CaMDH* as the most appropriate RGs (Table 4). Meanwhile, NormFinder ranked the genes as follows: *CaACT* > *CaMDH* > *CaUBQ2*, similar to the RefFinder tool. Delta-Ct identified the most reliable genes, starting with *CaACT*, followed by *CaUBQ2* and *CaMDH*. As observed in previous comparisons (Tables 2 and 3), BestKeeper differed considerably from the other tools, ranking the genes in this order: *CaEFA1* > *CaMDH* > *CaRPL39*. All tools agreed that the least optimal RGs for an experiment comparing ABA and GA_3_ treatments were *CaTUB* and *CaAP47*.

**Table 4:** Ranking of candidate reference genes according to stability values calculated by the geNorm, NormFinder, BestKeeper, Delta-Ct, and RefFinder algorithms, using Cq (Quantification Cycle) data from leaves of coffee plants under all treatments. M, mean expression stability value (geNorm); SV, stability value (NormFinder); SD, standard deviation of Cq values (BestKeeper); ΔCt, ΔCt stability value (comparative ΔCt method)

Gene	geNorm M	Rank	NormFinder SV	Rank	BestKeeper SD	Rank	ΔCt	Rank	RefFinder	General Rank
*CaACT*	0.214	1	0.107	1	0.858	5	0.633	1	1.495	1
*CaMDH*	0.318	3	0.193	2	0.767	2	0.657	3	2.632	2
*CaUBQ2*	0.302	2	0.221	3	0.857	4	0.650	2	2.913	3
*CaGAPDH*	0.214	1	0.222	4	0.874	6	0.669	4	3.130	4
*CaEFA1*	0.365	5	0.348	6	0.587	1	0.713	6	3.834	5
*CaRPL39*	0.341	4	0.342	5	0.845	3	0.697	5	4.401	6
*CaTUB*	0.530	6	0.928	7	1.145	7	1.118	7	7.000	7
*CaAP47*	0.887	7	1.914	8	1.530	8	1.957	8	8.000	8

### Reference genes validation

**Fig. 2:**
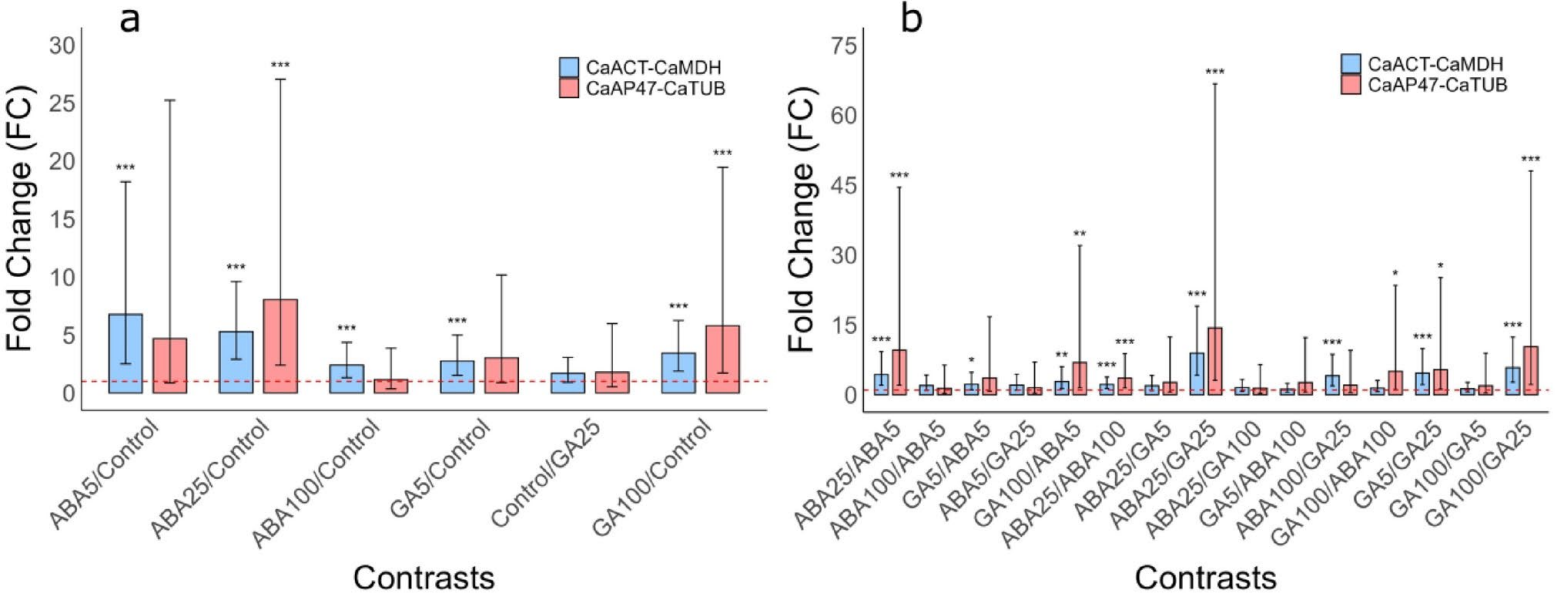
Validation of reference genes selection through normalization of *CaTFL1* expression in coffee leaves treated with ABA and GA_3_. Relative expression of the flowering-related gene *CaTFL1* determined using the two most stable RGs (*CaACT* and *CaMDH*, blue bars) and the two least stable RGs (*CaAP47* and *CaTUB*, red bars). (**a**) Contrasts between each hormonal treatment (ABA and GA_3_ at 5, 25, and 100 ppm) and the untreated control. (**b**) Pairwise contrasts among hormonal treatments and concentrations. Bar heights represent fold-change (FC) estimates obtained using the linear mixed model (LMM) approach, while black error bars indicate the corresponding 95% confidence intervals. The dashed red line represents the significance threshold (FC = 1); contrasts whose confidence intervals do not intersect this line were considered statistically significant. Asterisks indicate significance levels (*** for *P* < 0.001, ** for *P* < 0.01, and * for *P* < 0.05; *n* = 3 biological replicates)

To validate the RGs, the two most stable genes (*CaACT* and *CaMDH*) and the two least stable genes (*CaAP47* and *CaTUB*) were selected based on their rankings (Tables 2, 3 and 4) to normalize the expression data of the target gene, *TERMINAL FLOWER* (*CaTFL1*) (Fig. 2). The expression profile of *CaTFL1* varied notably depending on the RGs used. For example, when *CaACT* and *CaMDH* were used, *CaTFL1* expression was significantly higher in plants treated with ABA5 (7-fold, *p* < 0.001), ABA25 (5.5-fold, *p* < 0.001), ABA100 (2.5-fold, *p* < 0.001), GA5 (2.7-fold, *p* < 0.001), and GA100 (3.4-fold, *p* < 0.001) compared to control plants, with no significant difference observed in plants treated with 25 ppm of GA_3_ (Fig. 2a). Conversely, when the least stable genes (*CaAP47* and *CaTUB*) were used as references, *CaTFL1* expression increased significantly (*p* < 0.001) only in plants treated with 25 ppm of ABA (8-fold) and 100 ppm of GA_3_ (5.8-fold). In all cases, confidence intervals were wider when using the least stable genes (Fig. 2a).

When comparisons were made between treatments and concentrations, the expression of *CaTFL1* was higher in plants treated with 25 ppm of ABA (4.3-fold, *p* < 0.001), 5 ppm of GA_3_ (2.3-fold, *p* < 0.05), and 100 ppm of GA_3_ (2.8-fold, *p* < 0.01) compared to those treated with 5 ppm of ABA, using *CaACT* and *CaMDH* as RGs (Fig. 2b). Additionally, plants treated with 25 ppm of ABA exhibited higher expression of *CaTFL1* than those treated with 100 ppm of ABA (2.2-fold, *p* < 0.001) and 25 ppm of GA_3_ (8.9-fold, *p* < 0.001). Moreover, plants treated with 100 ppm of ABA and 5 ppm of GA_3_ exhibited higher expression of *CaTFL1*, showing a 4-fold increase (*p* < 0.001) compared to those treated with 25 ppm of GA_3_. Finally, plants treated with 100 ppm of GA_3_ presented higher expression of *CaTFL1* (5.8-fold, *p* < 0.001) compared to those treated with 25 ppm of GA_3_ (Fig. 2b).

Using the least stable pair of RGs, plants treated with 25 ppm ABA and 100 ppm GA_3_ exhibited higher expression of *CaTFL1* than those treated with 5 ppm ABA, with increases of approximately 10-fold (*p* < 0.001) and 7-fold (*p* < 0.001), respectively (Fig. 2b). Furthermore, plants treated with 25 ppm ABA showed a 1.8-fold higher expression of *CaTFL1* compared to those treated with 100 ppm ABA (*p* < 0.001) and a 3.8-fold increase compared to those treated with 25 ppm GA_3_ (*p* < 0.001). Unlike the results obtained with the most stable genes, using *CaAP47* and *CaTUB* as RGs revealed a difference between the ABA100 and GA100 treatments, with plants treated with 100 ppm GA_3_ showing significantly higher *CaTFL1* expression (5-fold, *p* < 0.05) than those treated with 100 ppm ABA. Finally, the expression of the target gene was higher in plants treated with 5 ppm GA_3_ (5.4-fold, *p* < 0.05) and in plants treated with 100 ppm GA_3_ (10.3-fold, *p* < 0.001) compared to plants treated with 25 ppm GA_3_. In general, the confidence intervals are wider when the least stable genes are used (Fig. 2b).

## Discussion

The accurate normalization of RT-qPCR data is contingent upon the selection of appropriate RGs [21, 23, 24, 39]. The knowledge about available RGs in coffee plants is limited, therefore, analyzing candidate RGs is especially important under different conditions. Since at least two RGs are needed to normalize qPCR data [40], it is essential to identify as many suitable genes as possible. In this work, eight potential RGs were evaluated in coffee leaf tissue treated with ABA and GA_3_ at three different concentrations. These genes have previously been used as normalizers in other plants [39, 41, 42] and in coffee [21, 23, 24].

According to the results obtained, it was observed that the eight genes (*CaACT*, *CaGAPDH*, *CaEFA1*, *CaTUB*, *CaAP47*, *CaMDH*, *CaRPL39*, and *CaUBQ2*) exhibited similar expression profiles with different Cq values (Fig. 1a). This is supported by the correlation analysis, where almost all genes showed high correlation coefficients (Fig. 1b). However, two genes (*CaAP47* and *CaTUB*) exhibited high variability (Fig. 1a) and low correlation, both between them and with other genes (Fig. 1b). This may indicate that these two genes were influenced by the treatments, since the other six genes displayed a similar expression pattern (Fig. 1b). This observation is significant because it suggests that the remaining six genes respond similarly across treatments, and the minor variation observed (Fig. 1a) could reflect inherent manipulation errors, such as pipetting. Therefore, considering that these six genes are housekeeping genes [10], the strong expression correlation (Fig. 1b) indicates that the analyzed samples were comparable, and any variation in a target gene would reflect genuine differences caused by the treatments. This initial observation from the raw Cq and correlation data was subsequently confirmed by the comprehensive stability analysis performed with the five algorithms (Tables 2, 3 and 4). However, geNorm ranked two genes equally as the most stable in all analyses (Tables 2, 3 and 4) because it identifies the best reference genes based on pairwise expression stability, as observed for *CaGAPDH*/*CaACT* (Fig. 1b; Tables 2 and 4) and *CaMDH*/*CaUBQ2* (Fig. 1b; Table 3). In contrast, BestKeeper consistently ranked *CaEFA1* as the most stable gene, whereas the other algorithms placed it in the 5th or 6th positions. This discrepancy arises from differences in the statistical approaches: while geNorm, NormFinder, and the Delta-Ct method assess stability through pairwise comparisons, BestKeeper evaluates the standard deviation (SD) and coefficient of variation (CV) of raw Cq values [15], favoring genes with the lowest absolute transcriptional variation, such as *CaEFA1* (Fig. 1a). By integrating the rankings from all algorithms [18], RefFinder reconciled these methodological differences and identified *CaACT* and *CaMDH* as the most reliable consensus RGs.

Due to the consideration of performing statistical analyses using the mixed linear model, as proposed by Steibel [36], which accounts for random effects, *CaMDH* and *CaACT* were selected to normalize the expression of *CaTFL1* in coffee plants treated with ABA and GA_3_. This model accounts for both fixed effects, corresponding to the hormonal treatments, and random effects, which represent biological and technical variability among samples. By modeling this random variance, the analysis provides more accurate estimates of gene expression differences and ensures that the selected RGs exhibit consistent expression across replicates, as observed for *CaMDH* and *CaACT*.

The *CaTFL1* gene is expressed in coffee leaf tissues, with one transcript featuring intron retention being accumulated during the flower induction period (February–April) and another transcript, without intron retention, being accumulated from June to August [43]. Heterologous expression studies confirm that transcript of *CaTFL1* without intron retention can delay flowering in Arabidopsis plants [43].

Interestingly, during the period from June to August, when the transcript without intron retention is abundant [43], ABA content in coffee leaves has been reported to increase [44], suggesting a potential influence of ABA on *CaTFL1* expression. This hypothesis was corroborated when *CaMDH* and *CaACT* were used as RGs, as all ABA concentrations increased *CaTFL1* expression compared to control plants (Fig. 2a). However, this result might not have been detected if *CaTFL1* expression had been normalized using the *CaAP47*-*CaTUB* pair as RGs, where only the ABA25 treatment showed statistical significance. This reliability is supported by the fact that the confidence intervals were considerably narrower when *CaMDH* and *CaACT* were used as RGs. According to Patino and Ferreira [45], the reliability of data increases when the confidence intervals are narrow, as this indicates that the data are more precise and less variable. Additionally, the width of confidence intervals is inversely proportional to the square root of the sample size (n) [45].

In the case of GA_3_ treatments, it has been suggested that the content of biologically active GA increases before the anthesis period, specifically in August [46]. Additionally, the expression of *CaTFL1* is negatively regulated by DELLA proteins [2], therefore, GA can induce *CaTFL1* expression. This was verified when *CaMDH* and *CaACT* were used as RGs, where all GA_3_ concentrations applied showed significantly higher *CaTFL1* expression compared to control plants (Fig. 2a). When *CaAP47* and *CaTUB* were used as RGs, only the treatment with 100 ppm of GA_3_ showed statistically significant *CaTFL1* expression, although with a wider confidence interval than that observed when *CaMDH* and *CaACT* were used as normalizers. Regarding the comparison of treatments and concentrations (Fig. 2b), normalization using the least stable RGs (*CaAP47* and *CaTUB*) produced larger effect estimates associated with wider confidence intervals, indicating greater uncertainty. In contrast, normalization with *CaMDH* and *CaACT* yielded more consistent and precise estimates.

The findings of this study underscore the context-dependent nature of RGs stability in coffee. For instance, *CaAP47*, one of the least stable genes under our hormonal treatments, has previously been reported as a reliable normalizer in roots subjected to drought stress [20] and in other tissues exposed to water stress conditions [21]. Similarly, *CaTUB*, which was also highly unstable in our study, has been identified as one of the most stable genes during somatic embryogenesis [24]. Conversely, genes such as *CaGAPDH* and *CaEF1α*, which showed moderate to high stability in our analysis, have also demonstrated stability under different abiotic stresses, such as nitrogen starvation, salinity, and heat [22].

These apparent discrepancies with the literature are not contradictory but rather highlight a fundamental principle of gene expression analysis: the stability of a RGs is strictly dependent on the specific experimental context. The instability of *CaAP47* and *CaTUB* in our study, for instance, can be explained by their putative involvement in the very hormonal pathways under investigation. The *CaTUB* gene, encoding a β-tubulin subunit, is a primary component of microtubules [47, 48]. The plant cytoskeleton is known to undergo significant dynamic reorganization in response to both abscisic acid (ABA), which modulates stomatal closure and stress responses, and gibberellins (GA), which are key regulators of cell elongation and division [27, 28, 30]. It is therefore highly plausible that the transcriptional regulation of tubulin genes is altered by these hormonal treatments, rendering them unsuitable as stable references in this context.

Similarly, *CaAP47* encodes a subunit of the clathrin adaptor protein complex, a key component of clathrin-mediated endocytosis (CME) [49, 50]. This process is critical for regulating hormone signaling by controlling the internalization, trafficking, and turnover of plasma membrane-bound receptors, including ABA importers and GA receptors [51]. As the application of these hormones can modulate the activity of this pathway to regulate cellular sensitivity, the transcriptional levels of its core components may be affected, thereby compromising their expression stability.

The superior stability of *CaACT* and *CaMDH* across ABA and GA_3_ treatments reflects their indispensable and tightly regulated roles in fundamental cellular homeostasis. Actin (*ACT*) encodes a major structural component of the plant cytoskeleton, essential for processes such as cytoplasmic streaming, cell division, and intracellular transport [52, 53]. Because structural integrity must be maintained during hormonal signaling and physiological transitions, actin transcript levels often remain highly uniform, a characteristic previously documented in *C. arabica* under other abiotic disruptions [23, 24]. On the other hand, malate dehydrogenase (*MDH*) catalyzes the interconversion of malate and oxaloacetate, playing a vital role in the tricarboxylic acid (TCA) cycle, malate-aspartate shuttle, and cellular redox balance [54]. Given that central carbohydrate metabolism and energy production are strictly buffered to ensure cell survival under physiological shifts induced by ABA and gibberellins, *CaMDH* exhibits minimal transcriptional fluctuations [55]. This physiological resilience reinforces why housekeeping pathways linked to basic structure and core energy metabolism serve as reliable anchors for transcript normalization in coffee plants subjected to growth regulator stimuli.

## Conclusions

This study successfully achieved its objective of identifying stable RGs for RT-qPCR analysis in coffee plants under hormonal treatments with ABA and GA_3_. Through a comprehensive evaluation using five distinct algorithms (geNorm, NormFinder, BestKeeper, DeltaCt, and RefFinder), this work highlights the importance of an integrated approach, rather than relying on a single method, to identify the most reliable candidates for a given experimental condition.

The results consistently identified *CaMDH* and *CaACT* as the most stable RGs across all analyses, making them highly suitable for normalizing gene expression data under these conditions. Importantly, the stability of these genes was validated specifically for coffee leaves collected during the reproductive developmental stage under ABA and GA_3_ treatments, and therefore should not be generalized to other tissues, developmental stages, or experimental conditions without prior validation. Conversely, *CaAP47* and *CaTUB* exhibited significant expression variability and are not recommended for use in this context. The critical importance of this selection was demonstrated in the validation analysis, where normalizing the expression of *CaTFL1*, a gene involved in floral induction, yielded accurate and precise results only when using the stable pair. This work provides a validated toolkit essential for future gene expression studies on the hormonal regulation of growth and development in coffee, ensuring greater accuracy and reliability in molecular research for this important crop.

## Supplementary Information

Below is the link to the electronic supplementary material.

Supplementary Material 1

Supplementary Material 2

Supplementary Material 3

## Data Availability

All data generated and analyzed during this study are included in this published article and its Supplementary Information file. No datasets were deposited in external public repositories.
