# Supplementary material for: Reference gene selection for gene expression analysis in Coffea arabica L. under ABA and gibberellin treatments
Source: Mol Biol Rep. 2026 Jul 31;53(1):1314. doi: 10.1007/s11033-026-12489-0 (PMC13427861; doi:10.1007/s11033-026-12489-0)
Supplement: Supplementary file 1 — Supplementary Material 1 [file 11033_2026_12489_MOESM1_ESM.pdf]

Reference Gene Selection for Gene Expression Analysis in *Coffea arabica* L. under ABA and Gibberellin Treatments

Lillian Magalhães Azevedo[1]; Robert Márquez-Gutiérrez[2]; Matheus Martins Daúde[3]; Horllys Gomes Barreto[3]; Renato Ribeiro de Lima[4]; Raphael Ricon de Oliveira[3]; Antonio Chalfun-Junior[2*].

[1]Central Laboratory of Molecular Biology (LCBM), Institute of Natural Science (ICN), Federal University of Lavras (UFLA), Lavras, Minas Gerais, Brazil.

[2]Laboratory of Plant Molecular Physiology, Plant Physiology Sector, Institute of Natural Science (ICN), Federal University of Lavras (UFLA), Lavras, Minas Gerais, Brazil.

[3]Laboratory of Molecular Analysis (LAM), Life Sciences Department, Federal University of Tocantins, Palmas, Tocantins, Brazil.

[4]Statistics Department, Federal University of Lavras (UFLA), Lavras, Minas Gerais, Brazil.

***Corresponding author:**
Antonio Chalfun-Junior
E-mail: chalfunjunior@ufla.br

Table S1 - Parameters for Minimum Information for Publication of Quantitative Real-Time PCR Experiments (MIQE) according to Bustin et al. (2009)

**Experimental design / Sample**	
Experimental group	Six-year-old plants of coffee (*Coffea arabica*)
Sample	Leaves
Sampling procedure	Immediately frozen in liquid nitrogen
Storage conditions / Time of storage	Freezer -80 °C / one month
**RNA extraction**	
Processing procedure	Grinding (mortar and pestle) in liquid nitrogen
Method	Organic extraction, according to De Oliveira et al., 2015.
RNA: DNA-free	TURBO DNA-*free*™ Kit (Ambion, Thermo Fisher Scientific) - Catalog number: AM1907
Nucleic acid quantification	Spectroscopy (NanoVue GE Healthcare)
RNA integrity	Agarose gel (1 %)
**Reverse transcription**	
Kit	High-Capacity cDNA Reverse Transcription Kit

	(Applied Biosystems, Thermo Fisher Scientific) - Catalog number: 4368814
Reaction conditions	25 °C 10’ / 37 °C 120’ / 85 °C 5’
Reverse transcriptase	MultiScrib^{TM} MuLV
Amount of RNA / Reaction volume	1 µg / 20 µL
Priming strategy	Random primers
Storage conditions of cDNA	Freezer -20 °C
**RT-qPCR**	
Target information	Table 1
Reaction conditions	Materials and Methods section
In silico	Primers were blasted using the BLAST tool at https://www.ncbi.nlm.nih.gov/
Empirical	Primer concentration of 1-2 µM (final concentration on the reaction) Annealing temperature: 60 °C
PCR efficiency	5-Fold dilution series of a mixed sample over at least five dilution points and verified to be higher than 85 % (E = 10−1/slope).
Linear dynamic range	Samples are situated within the range of the efficiency curves for each primer
No template control (NTC)	Cq and dissociation curve verification
**Data analysis**	
Specialist software	Qiagen Rotor Gene-Q Series software (version 1.7)
