# Supplementary material for: Reference gene selection for gene expression analysis in Coffea arabica L. under ABA and gibberellin treatments
Source: Mol Biol Rep. 2026 Jul 31;53(1):1314. doi: 10.1007/s11033-026-12489-0 (PMC13427861; doi:10.1007/s11033-026-12489-0)
Supplement: Supplementary file 3 — Supplementary Material 3 [file 11033_2026_12489_MOESM3_ESM.pdf]

Reference Gene Selection for Gene Expression Analysis in *Coffea arabica* L. under ABA and Gibberellin Treatments

Lillian Magalhães Azevedo[1]; Robert Márquez-Gutiérrez[2]; Matheus Martins Daúde[3]; Horllys Gomes Barreto[3]; Renato Ribeiro de Lima[4]; Raphael Ricon de Oliveira[3]; Antonio Chalfun-Junior[2*].

[1]Central Laboratory of Molecular Biology (LCBM), Institute of Natural Science (ICN), Federal University of Lavras (UFLA), Lavras, Minas Gerais, Brazil.

[2]Laboratory of Plant Molecular Physiology, Plant Physiology Sector, Institute of Natural Science (ICN), Federal University of Lavras (UFLA), Lavras, Minas Gerais, Brazil.

[3]Laboratory of Molecular Analysis (LAM), Life Sciences Department, Federal University of Tocantins, Palmas, Tocantins, Brazil.

[4]Statistics Department, Federal University of Lavras (UFLA), Lavras, Minas Gerais, Brazil.

***Corresponding author:**

Antonio Chalfun-Junior

E-mail: chalfunjunior@ufla.br

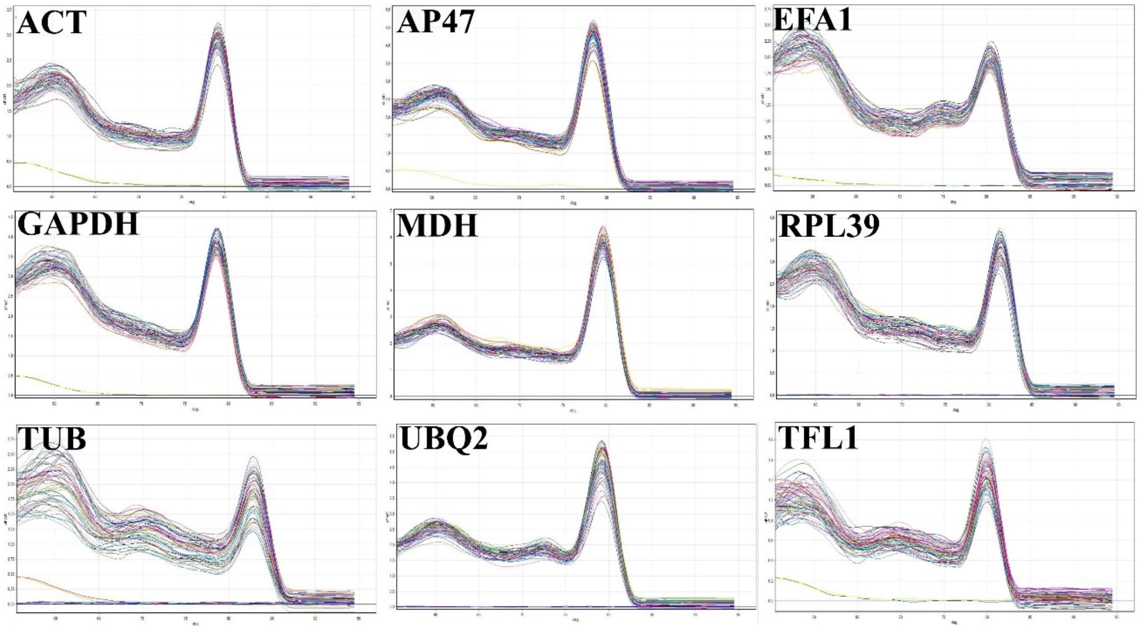

Fig. S1 Panel of dissociation (melting) curves for the primer pairs of the candidate reference genes evaluated in leaf tissues of *Coffea arabica* during the reproductive stage under ABA and GA_3 treatments. Each panel is identified by the corresponding gene name. The presence of a single peak for each primer pair confirms amplification specificity and the absence of nonspecific products.

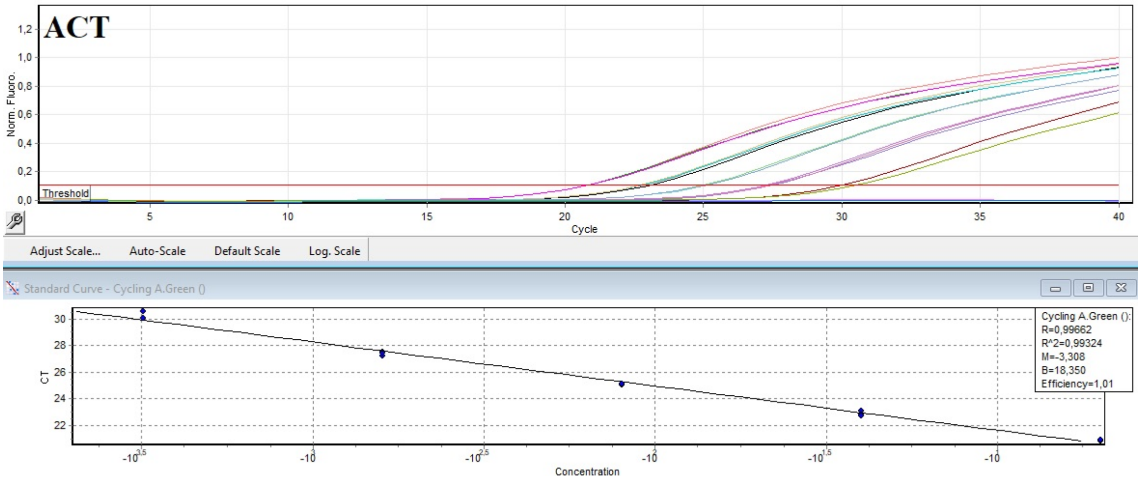
ACT
Norm. Fluoro.
Threshold
Cycle
Adjust Scale...
Auto-Scale
Default Scale
Log. Scale
Standard Curve - Cycling A.Green ()
CT
Concentration
Cycling A.Green ():
R=0,99662
R^2=0,99324
M=-3,308
B=18,350
Efficiency=1,01

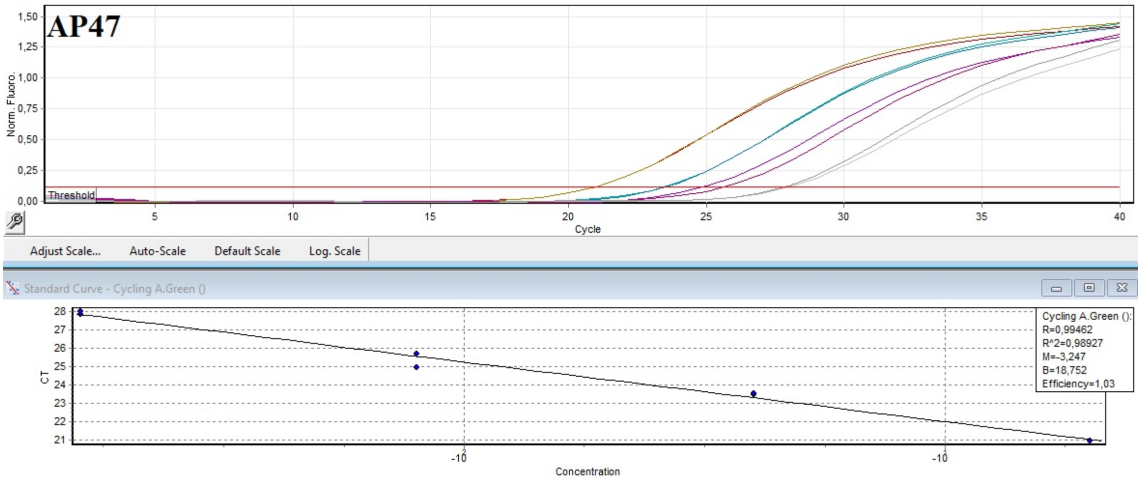
AP47
Norm. Fluoro.
Threshold
Cycle
Adjust Scale...
Auto-Scale
Default Scale
Log. Scale
Standard Curve - Cycling A.Green ()
CT
Concentration
Cycling A.Green ():
R=0,99462
R^2=0,98927
M=-3,247
B=18,752
Efficiency=1,03

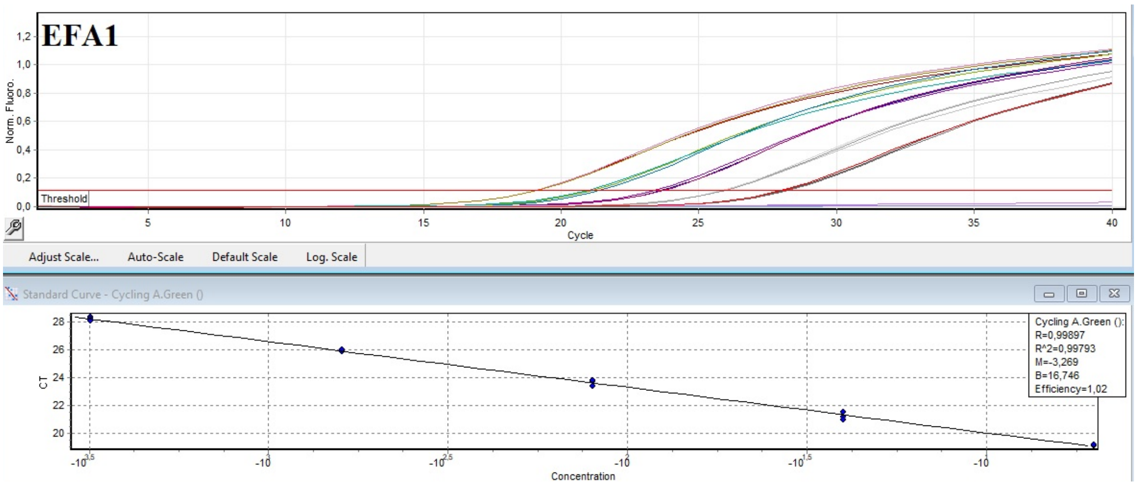
EFA1
Norm. Fluoro.
Threshold
Cycle
Adjust Scale...
Auto-Scale
Default Scale
Log. Scale
Standard Curve - Cycling A.Green ()
CT
Concentration
Cycling A.Green ():
R=0,99897
R^2=0,99793
M=-3,269
B=16,746
Efficiency=1,02

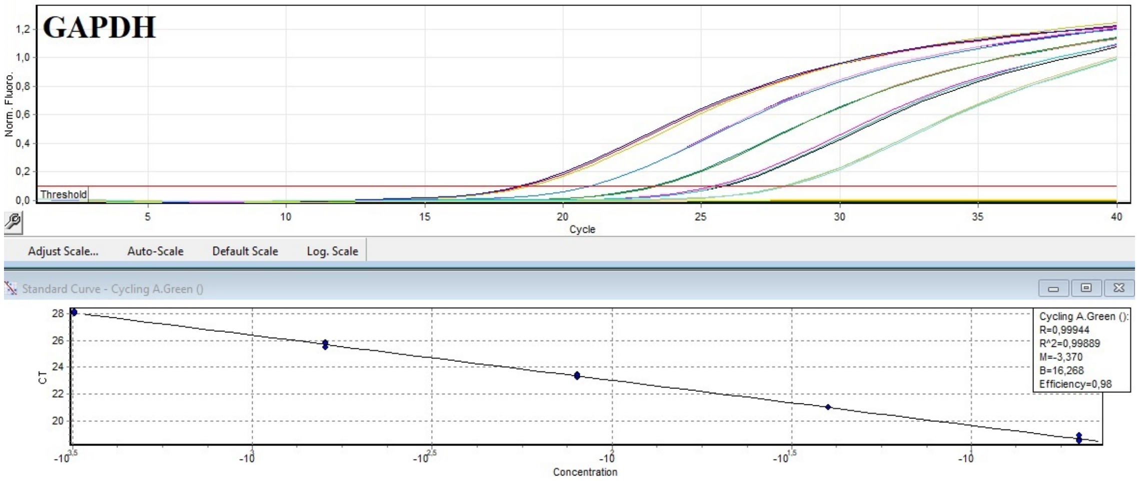
GAPDH
Norm. Fluoro.
Threshold
Cycle
Adjust Scale...
Auto-Scale
Default Scale
Log. Scale
Standard Curve - Cycling A.Green ()
CT
Concentration
Cycling A.Green ():
R=0,99944
R^2=0,99889
M=-3,370
B=16,268
Efficiency=0,98

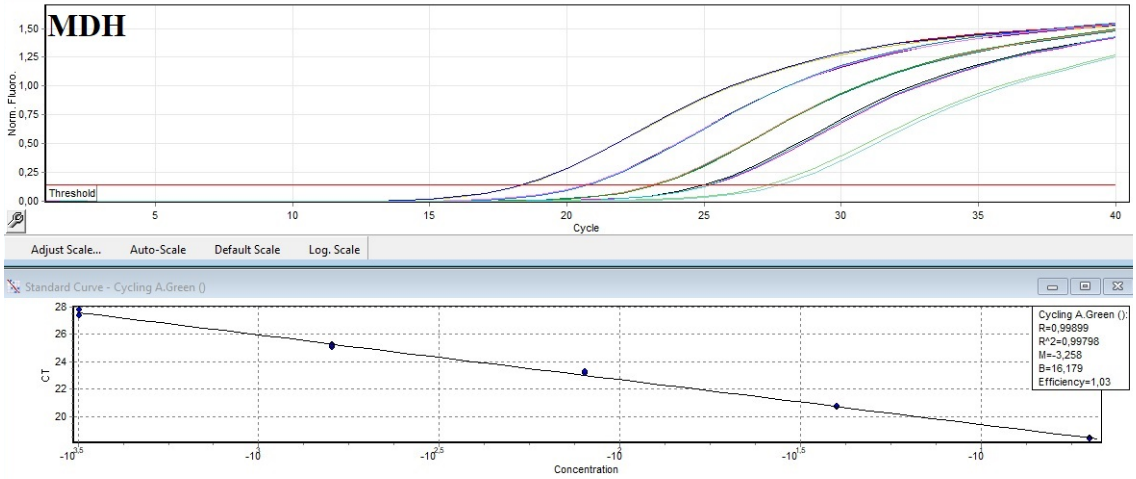
MDH
Norm. Fluoro.
Threshold
Cycle
Adjust Scale...
Auto-Scale
Default Scale
Log. Scale
Standard Curve - Cycling A.Green ()
CT
Concentration
Cycling A.Green ():
R=0,99899
R^2=0,99798
M=-3,258
B=16,179
Efficiency=1,03

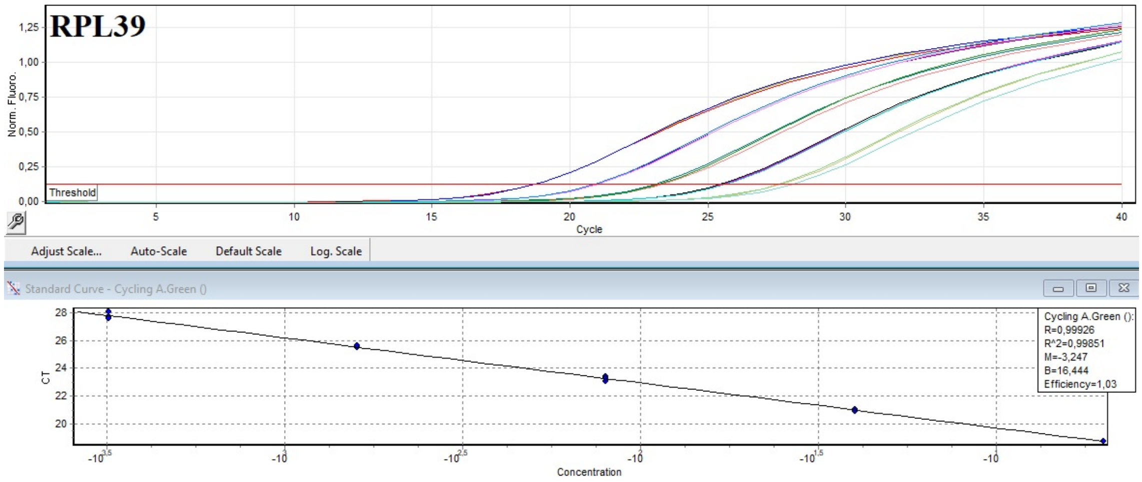
RPL39
Norm. Fluoro.
Threshold
Cycle
Adjust Scale...
Auto-Scale
Default Scale
Log. Scale
Standard Curve - Cycling A.Green ()
CT
Concentration
Cycling A.Green ():
R=0,99926
R^2=0,99851
M=-3,247
B=16,444
Efficiency=1,03

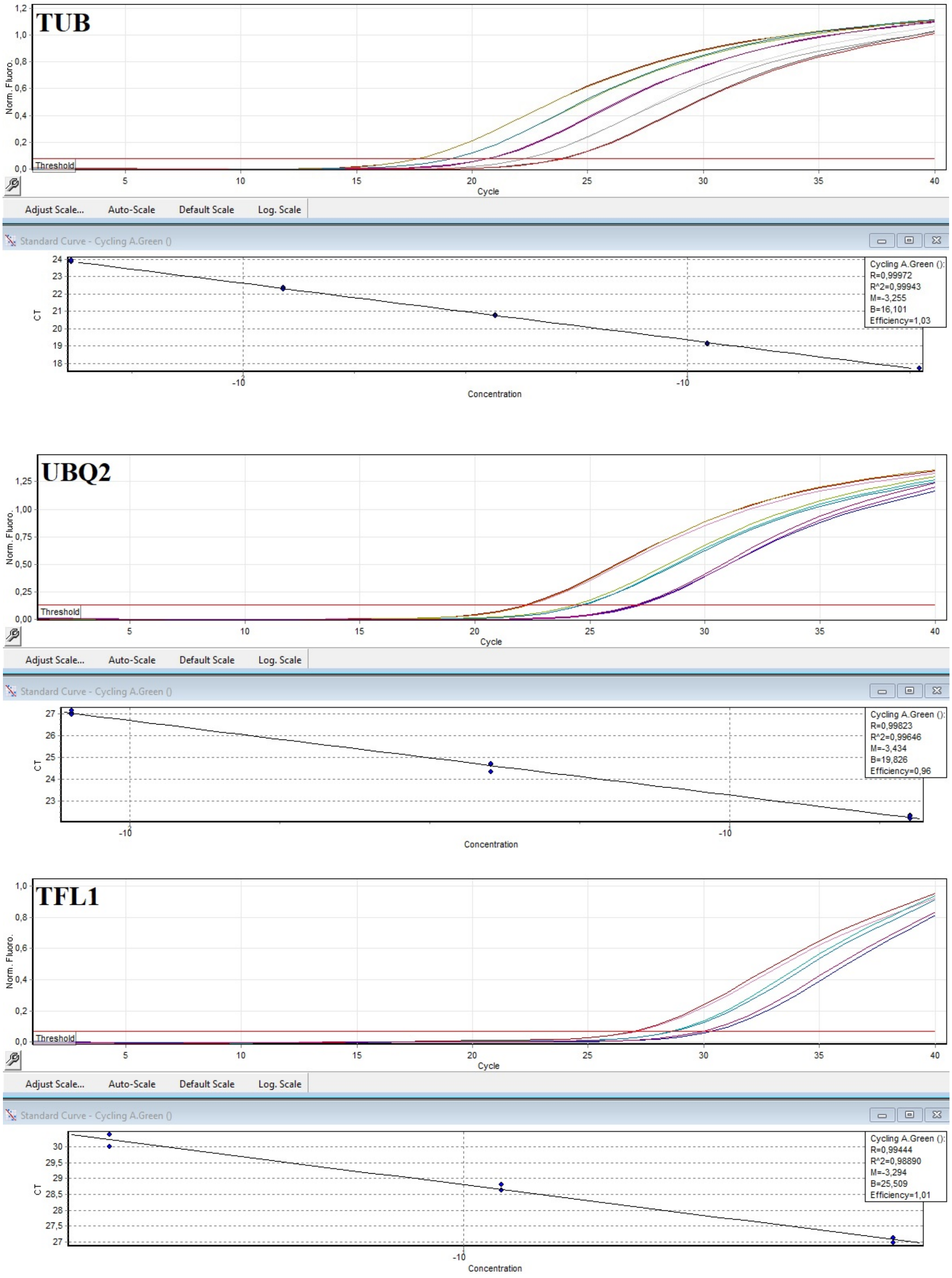

Fig. S2 Standard curves generated for the primer pairs of the candidate reference genes evaluated in leaf tissues of *Coffea arabica* during the reproductive stage under ABA and GA□ treatments. Each panel is identified by the corresponding gene name and displays the linear regression obtained from the serial cDNA dilutions used for assay validation. Amplification efficiency (E) and coefficient of determination (R^2) values are indicated for each primer pair, demonstrating the performance, linearity, and reliability of the RT-qPCR assays.
